# Illusory occlusion affects stereoscopic depth perception

**DOI:** 10.1038/s41598-018-23548-3

**Published:** 2018-03-28

**Authors:** Zhimin Chen, Rachel N. Denison, David Whitney, Gerrit W. Maus

**Affiliations:** 1 Department of Psychology, University of California Berkeley, Berkeley, CA USA; 2 Department of Psychology and Center for Neural Science, New York University, New York, NY USA; 3 Vision Science Program and Helen Wills Neuroscience Institute, University of California, Berkeley, Berkeley, CA USA; 4 Psychology Programme, School of Social Sciences, Nanyang Technological University, Singapore, Singapore

## Abstract

When occlusion and binocular disparity cues conflict, what visual features determine how they combine? Sensory cues, such as T-junctions, have been suggested to be necessary for occlusion to influence stereoscopic depth perception. Here we show that illusory occlusion, with no retinal sensory cues, interacts with binocular disparity when perceiving depth. We generated illusory occlusion using stimuli filled in across the retinal blind spot. Observers viewed two bars forming a cross with the intersection positioned within the blind spot. One of the bars was presented binocularly with a disparity signal; the other was presented monocularly, extending through the blind spot, with no defined disparity. When the monocular bar was perceived as filled in through the blind spot, it was perceived as occluding the binocular bar, generating illusory occlusion. We found that this illusory occlusion influenced perceived stereoscopic depth: depth estimates were biased to be closer or farther, depending on whether a bar was perceived as in front of or behind the other bar, respectively. Therefore, the perceived relative depth position, based on filling-in cues, set boundaries for interpreting metric stereoscopic depth cues. This suggests that filling-in can produce opaque surface representations that can trump other depth cues such as disparity.

## Introduction

The human visual system has a variety of mechanisms for extracting depth information from the environment. How the available cues to depth are combined to produce a coherent representation of the three-dimensional structure of a scene is critical for understanding the ability to perceive depth. Binocular disparity, the difference between the images on the retinae of the two eyes due to the horizontal distance between the eyes, is one important depth cue^1^. It is a metric depth cue, as it provides information about the absolute distance in depth between two objects. A number of studies have proposed that various types of metric depth cues are combined optimally in a probabilistic framework^2–6^. Ordinal cues, such as occlusion, convey information about the ordering of objects in depth, but not about the metric depth of objects^7^. The combination of metric and ordinal depth cues poses a computational challenge to probabilistic cue integration models, because these two cue types cannot be measured in the same units^5^. When an ordinal cue is congruent with other depth cues, it can help to disambiguate other ambiguous cues, such as in the kinetic depth effect^8^. When ordinal and metric cues conflict, however, the resolution can be complex: one cue may become dominant and suppress the other, or the two types of cues may be interpreted as from distinct objects^9^.

Occlusion and disparity are two prevalent ordinal and metric cues, respectively, and they have been pitted against each other in the laboratory for studying cue combination. Occlusion cues, such as T-junctions, L-junctions and bounding contours, can convey unambiguous relative position information about the two objects, so that when occlusion and stereoscopic cues conflict and give incompatible depth information, occlusion usually overrides disparity. For example, a pseudoscope—an optical instrument which swaps the left and right eye’s views and thus presents the visual system with reversed disparities—does not cause viewers to perceive the scene reversed in depth. Instead, objects in the scene retain their apparent relative positions consistent with occlusion cues. Surprisingly, most viewers do not report any unusual percepts^10^. In other situations, when the conflict between occlusion and disparity is relatively small, the visual system reconciles conflicting occlusion and disparity signals by perceiving transparent^11^, broken^12^, or bending surfaces^13,14^. Depth discrimination between two vertically aligned bars that have different physical disparities can be impaired when the bars are likely to be perceptually completed behind an occluding horizontal bar^15^. Figure-ground assignments can also bias the perception of metric stereoscopic depth^16–19^. These cases indicate that representations of an occluding surface can influence depth judgments. However, in all these cases there are bottom-up retinal cues (e.g., contour junctions or bounding contours) signaling the presence of occluding surfaces. Whether cues to occlusion from perceived global configurations alone can interact with unambiguous disparity cues remains unknown.

To determine whether retinal cues are necessary for occlusion to dominate depth perception, we employed “illusory occlusion”, in which one object is seen in front of and occluding another object, although no contour junctions or bounding contours are present on the retina^20,21^. When two differently coloured bars forming a cross are viewed monocularly and placed such that their intersection point falls inside the retinal blind spot, the visual system does not receive bottom-up information about the relative depth position of the two bars at the intersection point. Rather than fusion or the appearance of transparency, either bar was found to be perceptually filled in and seen in front of the other. This ambiguous global depth configuration is resolved by a rivalry process, which leads to perception of an unambiguous interpretation of one bar being occluded by the other, and over time the perceived depth ordering alternates^21^.

Here we use this phenomenon of illusory occlusion to investigate how occlusion in the absence of retinal cues is integrated with an unambiguous binocular disparity signal. We used the blind spot as a tool to generate illusory occlusion, although the phenomenon may also appear in other retinal locations or under different scenarios, such as artificial scotomata. We report a series of three experiments showing that illusory occlusion without retinal depth cues is a strong cue to depth that influences even non-ambiguous metric cues from physical retinal disparity. We conclude that filling-in at the blind spot produces opaque surface representations that provide contributions to other perceptual processes, including depth estimation.

## General Methods

### Participants

All experimental procedures were approved by and conducted in accordance with the guidelines and regulations of the University of California at Berkeley Institutional Review Board. Ten observers (4 male, 6 female, mean age: 24.5, age range: 20–34 years), including two of the authors, participated in the experiment after giving informed consent in accordance with the IRB guidelines of the University of California at Berkeley. Four observers were excluded from completing the study because of poor stereoscopic discrimination in Experiment 2 A; they were near chance (~50%) when discriminating stimuli at the maximum crossed disparity used. Data from the remaining six participants was used in the following analyses. Only five out of six observers participated in Experiment 3B, because one observer dropped out before completion of the study. All observers had normal or corrected-to-normal visual acuity and were experienced with psychophysical experiments.

### Apparatus

All stimuli were generated using Matlab (MathWorks) and Psychtoolbox 3^22,23^, running on an Apple Macintosh computer. Stimuli were displayed on two 21-inch CRT monitors (Sony Trinitron Multiscan G520) with a spatial resolution of 1280 × 1024 pixels and a frame rate of 75 Hz. Each eye of the observer viewed only one monitor at a viewing distance of 42 cm using a haploscopic setup. The two monitors were positioned to the left and right of the observer with their screens facing each other. Observers viewed the screens through two mirrors placed at 45° angles in front of the eyes. Head position was stabilized with a chin-and-forehead rest. Responses were recorded with mouse clicks and keyboard button presses.

### Stimuli

**Figure 1:**
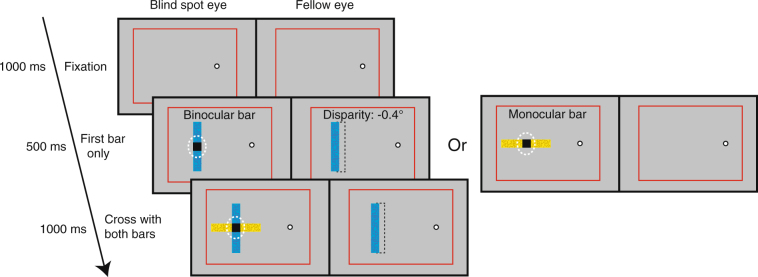
Stimuli and trial sequence of Experiment 1. Each pair of panels represents a pair of left- and right- eye images. Either a “binocular bar” or a “monocular bar” was presented for 500 ms, followed by both bars for 1000 ms. The binocular bar was presented to both eyes, with its image in the blind spot eye reaching through the blind spot and its image in the fellow eye shifted for a crossed disparity of −0.4°. The monocular bar was presented reaching through the blind spot area and was seen as a complete object due to perceptual filling-in when presented alone. Observers were asked to report which bar was seen in front of the other. The white dashed circle represents the blind spot area of the blind spot eye (not to scale). The black dashed rectangles in the fellow eye show the positions of the corresponding images presented in the blind spot eye. Red frames on each screen provided vergence cues.

The visual stimuli were rectangular bars with a length of 12° and a width of 1.7°. The bars were distinguished by having different but isoluminant colors, either yellow or blue (52.9 cdm^−2^). They were presented on a darker gray background (18.1 cdm^−2^) at 50% Michelson contrast. One of the bars (the “monocular bar”) was presented monocularly only to the blind spot eye (Fig. 1: yellow bar). The other bar (the “binocular bar”) was binocularly presented to both eyes with the image in the fellow eye shifted horizontally to induce disparity (Fig. 1: blue bar). The binocular bar had a fixed disparity of −0.4° (negative value = crossed disparity, appearing closer than the plane of the fixation point). In the blind spot eye, images of both bars were centered on the measured blind spot area. In the fellow eye, the image of the binocular bar was centered on the corresponding area and was completely visible to observers. In order to help observers utilize the disparity cues, 150 dots (diameter 0.2°) were randomly spread over the surface of each bar. The dots had the same hue as the bar, either all yellow or all blue, but were reduced in luminance to 24.0 cdm^−2^. During pilot experiments, we found that the monocular bar was rarely filled in across the blind spot and seen dominant when presented together with the binocular bar, which may be because interocularly unpaired regions are subject to suppression^24^. In order to make the monocular bar more likely to be perceived as occluding the binocular bar, it was oriented horizontally so that part of it would be closer to the observer’s fovea. The binocular bar was oriented vertically. In addition, the monocular bar moved up and down continuously at a rate of 0.91 cycles per second. The intersection of the bars was always well within the measured blind spot area: the top- and bottom-most positions of the bar’s trajectory were on average 1.38° (*SEM* 0.27°) from the measured upper and lower blind spot boundaries. A black square covering the intersection of the bars was presented to the blind spot eye. If at any time observers became aware of this black square, they were instructed to abort the trial and refixate on the fixation dot. The percentage of trials aborted due to instable fixation was 1.13% (SD 1.19%), and in no participant more than 3% were excluded. In different experiments and conditions (see below), the bars were shown either in isolation or combined (a monocular bar and a binocular bar simultaneously forming a cross).

The fixation point was a black (0.42 cdm^−2^) annulus with a white (115.6 cdm^−2^) center. It was 0.5 degrees of visual angle in diameter and was placed 7.7° to the left or right of the center of the screen, visible to both eyes. In addition, a centered red rectangular frame (length 30°, width 25°) was presented to both eyes to help observers fuse the images and minimize vergence eye movements. The observer was instructed to maintain fixation while paying attention to the stimuli in the periphery.

### Overview of procedure

Before the experiments, we determined each observer’s sighting eye dominance with the ‘hole in the hand test’ and the Porta test variant^25^. We then measured the center and the extent of each observer’s blind spot for the dominant eye using the same procedure as in Chen *et al*.^21^, which is similar to previous studies^26,27^. A small flickering square cursor (side length 0.4°) was presented only to the blind spot eye. Observers were instructed to slowly move the cursor from different directions into the blind spot until the pointer became completely invisible. They then moved the cursor back and forth to ensure that the cursor was just inside the blind spot, and clicked the mouse to indicate this position. Six defining positions along the blind spot boundary were mapped three times and the mean of the three measurements was used to define the blind spot boundary. First, we allowed the cursor to move along an invisible horizontal line that went through the center of the screen and the fixation spot, and measured the two boundaries of the blind spot on the horizontal meridian. Next, we allowed the cursor to move along a vertical line that passed through the center between the two horizontal boundaries just obtained, and measured the two vertical boundaries and the vertical diameter of the blind spot. Finally, we allowed the cursor to move along a line passing through the vertical center of the blind spot and measured another two horizontal boundaries (which defined the horizontal diameter). The intersection between the horizontal and vertical diameters defined the blind spot center. This blind spot measurement procedure served the experimental purpose, ensuring that the center of the stimulus cross always remained within the blind area.

In Experiment 1, we verified our method to manipulate which bar, monocular or binocular, was seen in front as the occluder. In Experiment 2, we measured the baseline depth estimates of the two bars: we separately measured the perceived depth of the binocular bar presented at a fixed retinal disparity (Experiment 2A), and the monocularly presented bar (Experiment 2B). In Experiment 3, we then measured how the perceived depth of the binocular bar (Experiment 3A) or the monocular bar (Experiment 3B) was influenced by the presence of a task-irrelevant monocular or binocular bar, depending on the perceived relative depth of the two bars. Experiment 2A was always carried out first to establish that observers could readily perceive depth from disparity. Eligible observers were then tested in other experiments in the order of 2B, 1 and 3.

## Experiment 1: Test of illusory occlusion

### Methods

The purpose of this experiment was to test a method of manipulating illusory occlusion. We investigated whether a monocular bar that is perceptually completed through the blind spot by filling-in could be seen as occluding a binocularly presented bar, when the two bars crossed in the blind spot region of the eye viewing the monocular bar. Further, we tested whether the perceptual dominance of one bar over the other could be manipulated by delaying the onset of one of the two bars. We hypothesized that the bar that appeared later in time would dominate and appear to be occluding the other bar. We predicted that, similar to interocular flash suppression^28,29^, the transient onset of the monocular bar would suppress the binocular bar in the corresponding portion of the visual field and bias the visual system to fill in the monocular bar. This process would generate illusory occlusion.

In one condition, we presented the binocular bar (at a fixed disparity of −0.4°) for 500 ms, and then added the monocular bar to the display. Both bars formed a cross that was centered in the blind spot area and remained visible for an additional 1000 ms (Fig. 1). Observers were asked to judge in a 2-alternative forced-choice (2AFC) task whether the horizontal bar (the monocular bar) or the vertical bar (the binocular bar) appeared in front. After the stimulus presentation, observers indicated their decision by pressing one of the two buttons on a computer keyboard under no time pressure. In the other condition, the temporal order of presentations was reversed: we presented the monocular bar alone first for 500 ms, and then added the binocular bar to the display to form a cross for an additional 1000 ms. Colors of the bars were different and randomly assigned on each trial. Experiment 1 had 20 trials for each of the two presentation orders. Both conditions were randomly interleaved.

### Results

**Figure 2:**
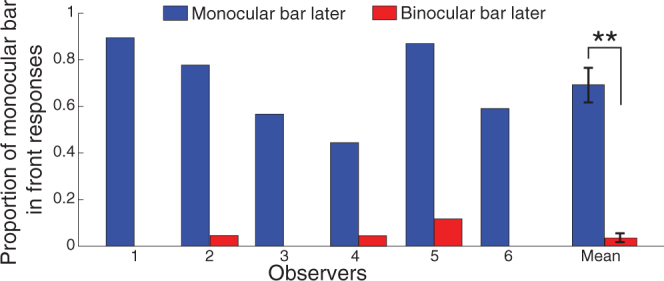
Results of Experiment 1: test of illusory occlusion. Proportion of trials in which the monocular bar was seen in front relative to the binocular bar under the two presentation order conditions (**p < 0.001). The error bars show ± 1 standard error of the mean.

We found that a monocular object filled-in through the blind spot with a delayed onset was seen as in front of and occluding a binocularly viewed object, even when the binocular object was presented as closer than the fixation plane using disparity cues, as predicted based on interocular flash suppression^28,29^. These findings verify that varying the temporal presentation order can change the perceived relative depth position of the bars. Results for all 6 individual observers and the group mean are shown in Fig. 2. In the condition in which the monocular bar was presented later, observers reported seeing the monocular bar as in front of, or occluding, the binocular bar in 69% ± 7.47% (mean ± SEM) of trials. In the condition in which the binocular bar appeared later, observers rarely reported seeing the monocular bar as in front, doing so only in 3.48% ± 1.89% (mean ± SEM) of trials. The probability of an illusory occlusion (the monocular bar seen in front of the binocular bar) was significantly increased by presenting the monocular bar later (two tailed paired t-test, *t*(5) = 9.27, *p* = 0.00025, Cohen’s d = 4.92)

## Experiment 2: Baseline perceived depth

### Methods

**Figure 3:**
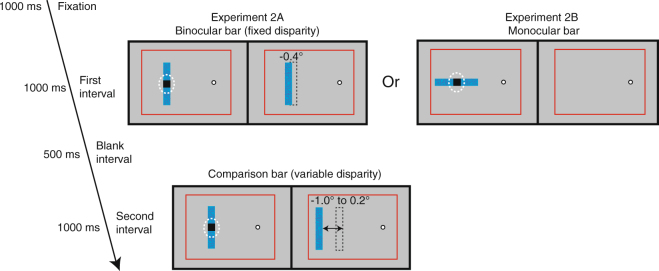
Stimuli and trial sequence of Experiment 2. A 2IFC task was used to measure the baseline perceived depth of the binocular bar and the monocular bar. Either a “binocular bar” (Experiment 2A) or a “monocular bar” (Experiment 2B) was presented for an interval of 1000 ms. After a blank interval of 500 ms, a “comparison bar” with variable disparity was presented for 1000 ms. Observers were asked to report whether the bar in the first or the second interval was seen closer in depth. The “comparison bar” was binocularly presented in a similar way as the “binocular bar” but with its image in the fellow eye shifted for a disparity between −1° and 0.2° (in steps of 0.2°). Other display elements as in Fig. 1.

In Experiment 2, we employed a 2-interval forced-choice (2IFC) paradigm to measure the perceived depth of the binocular bar (Experiment 2A) and the monocular bar (Experiment 2B), when either bar was presented alone (Fig. 3). To do this, we presented a second binocular bar as the “comparison bar” in a separate interval, and varied its disparity between −1° and 0.2° in steps of 0.2° using the method of constant stimuli. Like the binocular bar, the comparison bar was always oriented vertically with its image in the blind spot eye centered on the blind spot area.

In Experiment 2A, we presented the binocular bar at a fixed disparity of −0.4° for 1000 ms. After an inter-stimulus interval (ISI) of 500 ms, we presented the comparison bar for another 1000 ms. Observers were asked to judge in which interval the bar appeared closer in depth to them. On a given trial, both bars in the two intervals had the same color, which was either yellow or blue and randomized across trials. Experiment 2B employed the same procedure as Experiment 2A except that we presented the monocular bar in the first interval. Observers were asked to respond whether the bar in the first interval (the monocular bar) or in the second interval (the comparison bar) appeared closer to them. Observers indicated their decision by pressing the corresponding button under no time pressure. Experiment 2A and 2B consisted of 140 trials each, 20 repetitions ×7 disparities of the comparison bar.

### Data Analysis

**Figure 4:**
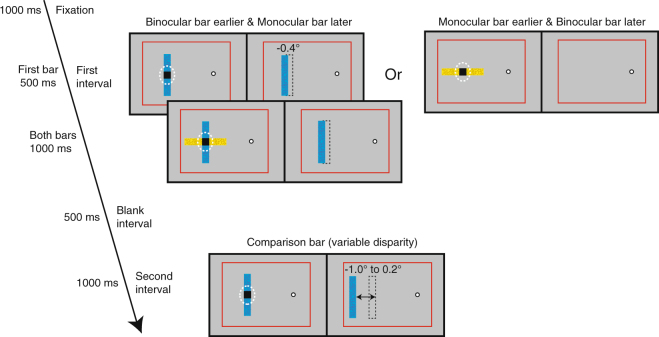
Stimuli and trial sequence of Experiment 3. A 2IFC task was used to measure the perceived depth of the binocular bar and the monocular bar with illusory occlusion. Either a “binocular bar” or a “monocular bar” was presented for 500 ms, followed by the other bar for another 1000 ms. After a blank interval of 500 ms, a “comparison bar” with variable disparity was presented for 1000 ms. Only the task was different in Experiment 3A and 3B. In Experiment 3A, observers were asked in which interval the vertical bar appeared closer in depth to them. In Experiment 3B, observers were asked whether the horizontal bar in the first interval or the vertical bar in the second interval appeared closer in depth to them. Other display elements as in Fig. 1.

Cumulative Gaussian functions were fitted to the proportion of “closer” responses as a function of disparity of the comparison bar for each observer. We determined points of subjective equality (PSE), the points at which an observer was equally likely to report a bar as “closer” or “farther” than the comparison bar. The PSE measures the perceived depth of the binocular bar or the monocular bar when they were presented alone (Experiment 2; Fig. 3) and in different contexts (Experiment 3; Fig. 4).

### Results

**Figure 5:**
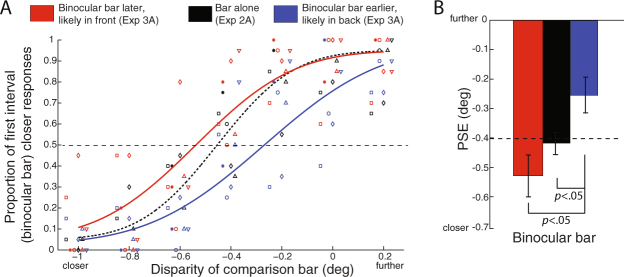
Perceived depth of the binocular bar in Experiment 2A and 3A. (**A**) Aggregate psychometric functions for 6 observers when the depth of the binocular bar was judged relative to the comparison bar in a 2IFC task. The binocular bar was either presented alone (black) or presented after (red) or before (blue) a task-irrelevant monocular bar. Symbols show individual data points from individual observers (jittered horizontally to avoid clustering). (**B**) Mean and SEM of disparity PSEs from psychometric functions fitted to individual observers’ data, which show perceived depth of the binocular bar under three conditions.

**Figure 6:**
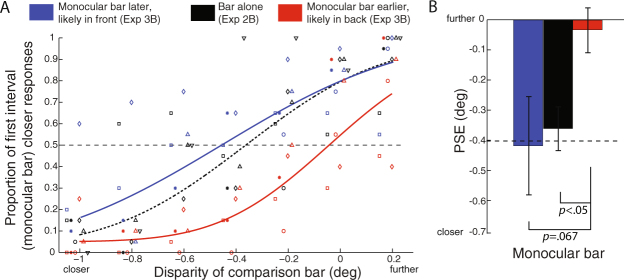
Perceived depth of the monocular bar in Experiment 2B and 3B. (**A**) Aggregate psychometric functions for 6 observers when the depth of the monocular bar was compared with the comparison bar. (**B**) PSEs (mean and SEM) of the monocular bar under three conditions. The error bars show ± 1 SEM. Other conventions as in Fig. 5.

We measured the baseline depth at which a monocular bar reaching through the blind spot and a binocular bar with a fixed, crossed disparity are perceived when the bars are presented in isolation. Data from all 6 individual observers and psychometric functions fitted to the mean proportion of responses across all observers are shown in Fig. 5 for the binocular bar and in Fig. 6 for the monocular bar (black symbols, lines, and bars).

In Experiment 2A (Fig. 5), when the binocular bar was presented alone, observers perceived its depth accurately (PSE = −0.42°, SEM = 0.038°), approximately at its physical disparity of −0.40° (no difference from physical: *t*(5) = −0.40, *p* = 0.70, Cohen’s d = −0.23). In Experiment 2B (Fig. 6), when the monocular bar was presented alone, observers perceived its depth to be equivalent to a binocular bar with crossed disparity (PSE = −0.36°, SEM = 0.074°), which is significantly closer in depth than an object of zero disparity (*t*(5) = −4.9, *p* = 0.0045, Cohen’s d = −2.8).

## Experiment 3: Test of perceived depth under illusory occlusion

### Methods

The purpose of this experiment was to investigate whether the apparent depth of a bar – either a filled-in bar in the blind spot or a binocular bar with disparity information – was affected by the perceived relative depth positions of the two bars. We manipulated the perceptual dominance of the binocular bar over the monocular bar by manipulating their onset asynchrony as established in Experiment 1. The monocular bar either appeared after the binocular bar and was therefore more likely to be seen occluding the binocular bar, or vice versa. We used a 2IFC paradigm and the method of constant stimuli to measure the perceived depth of the binocular bar (Experiment 3A) and the monocular bar (Experiment 3B) under these two experimental conditions (Fig. 4). Note that the bar stimuli observers were judging under both conditions were identical, and only the presentation timing varied.

After a fixation period of 1000 ms, we presented either the binocular bar or the monocular bar for 500 ms and then both bars together as a cross for 1000 ms. Both temporal presentation order conditions were randomly interleaved across trials. After an ISI of 500 ms, we then presented the comparison bar for 1000 ms. In Experiment 3A, observers were asked to judge in a 2IFC task whether the vertical bar in the first interval (the binocular bar) or the comparison bar in the second interval was closer to them. The horizontal monocular bar in the first interval was irrelevant to the task. In Experiment 3B, the same stimuli were presented, but observers were asked to judge whether the horizontal monocular bar in the first interval or the comparison bar in the second interval was closer to them. The vertical binocular bar in the first interval was irrelevant to the task. Colors of the monocular bar and the binocular bar were different and randomized between trials. The comparison bar had the same color as the binocular bar and was always oriented vertically, allowing it to have the same type of disparity signals as the binocular bar. Experiment 3A and 3B both contained 280 trials, 20 repetitions ×7 disparities of the comparison bar ×2 temporal presentation order conditions.

### Results

Perceived stereoscopic depth was affected by the apparent relative depth position from illusory occlusion. In Experiment 3A, we measured the perceived depth of the binocular bar (Fig. 5). When the monocular bar appeared later and the binocular bar was more likely to be seen as occluded by the monocular bar, observers’ mean PSE for the binocular bar was −0.25° (SEM = 0.062°), significantly farther away in depth than its baseline perceived depth when it was presented alone in Experiment 2A, *t*(5) = 3.9, *p* = 0.012, Cohen’s d = 1.3. When the binocular bar appeared later and was more likely to be seen as occluding the monocular bar, observers perceived it at a PSE disparity of −0.53° (SEM = 0.070°) and as significantly closer in depth than when the monocular bar appeared later, *t*(5) = −2.7, *p* = 0.042, Cohen’s d = −1.7, although not significantly closer than its baseline perceived depth (*t*(5) = −1.3, *p* = 0.26, Cohen’s d = −0.80).

The perceived depth of the monocular bar, which produced no retinal input near its intersection with the binocular bar, was also influenced by the perceived relative depth position (Experiment 3B; Fig. 6). When the binocular bar appeared later and was more likely to be seen as occluding the monocular bar, observers’ mean PSE for the monocular bar was −0.026° (SEM = 0.070°), significantly farther away than its baseline perceived depth (*t*(4) = 3.2, *p* = 0.025, Cohen’s d = 2.0). When the monocular bar appeared later and was more likely to be seen as occluding the binocular bar, observers’ mean PSE for the monocular bar was −0.40° (SEM = 0.16°), which was not significantly different from its baseline perceived depth in Experiment 2B (*t*(4) = −1.1, *p* = 0.34, Cohen’s d = −0.61), and also not significantly different from its perceived depth when the monocular bar appeared first (*t*(4) = 2.3, *p* = 0.067, Cohen’s d = 1.3).

### Data availability

All relevant data are available from the authors.

## Discussion

We developed a method to manipulate illusory occlusion and found that it can integrate with physical binocular disparity to influence perceived depth. A monocular and binocular stimulus intersected in the blind spot, resulting in a situation with no retinal occlusion cues and potentially ambiguous relative depth positions. Experiment 1 showed that the monocular stimulus was much more likely to perceptually dominate over the binocular stimulus and be seen closer in depth order when it had the more recent onset. Experiments 2 and 3 showed that the perceived metric depth of the binocular stimulus changed depending on its perceived relative depth position with the monocular stimulus: the stereoscopic object appeared farther away when it was seen occluded by the filled-in monocular stimulus, and it appeared closer in depth when it was seen as the occluder. The perceived depth of the monocular stimulus also depended on whether it was seen as in front of or behind the binocular stimulus, which might be due to the fact that the stereoscopic depth of interocularly unpaired areas are fundamentally ambiguous and influenced by the depth of adjacent unambiguous areas^30–32^. However, it is striking that the binocular bar’s depth was perceived differently in different contexts, because it had a physical, unambiguous disparity. All effects are based on comparisons of the same retinal stimuli (a horizontal monocular bar and a vertical binocular bar) in different contexts, ruling out potential confounds such as effects of different bar colours, orientations, or the presence of unpaired monocular signals^33^.

These experiments demonstrate that binocular disparity information can be quantitatively integrated with a filled-in illusory occlusion cue. Importantly, the occlusion cue was not defined by retinal contour junctions or bounding contours, but was internally generated by the blind spot filling-in process. The binocular object is perceived farther or closer in depth in its entirety according to the perceived relative depth between objects. These observations confirm the visual system’s bias for filling in opaque rather than transparent surfaces within the blind spot^21^. Here we demonstrate further that this filled-in surface has a functional representation that is able to influence other perceptual processes, similar to a physical surface.

We tested the perceived depth of either bar in isolation in Experiment 2. We removed the intersection of the cross pattern by presenting the horizontal and vertical stimuli in sequence. This has the advantage of leaving the retinal image of each bar unchanged, compared to other possibilities such as presenting the cross with the central intersection part deleted. We found in Experiment 2B that the monocular bar was best matched perceptually to a binocular object with a crossed disparity. As a monocularly viewed object does not carry interocular disparity information, we expected that it would be perceived in the fixation plane because of the vergence cue provided by the adjacent fixation point^34,35^. The non-zero perceived depth might be due to our choice of disparity range of comparison stimuli, which centered around the crossed disparity used for the binocular bar used in Experiment 2A. Another possibility is that the monocular bar is perceived at the distance of the horopter, which curves towards the observer in the far periphery; this curvature would place the monocular bar closer than the fixation plane^36^. These possibilities might have caused the monocular bar to be best matched to a binocular comparison bar with crossed disparity. However, the bias in baseline perceived depth does not affect the crucial comparisons in Experiment 3, where the perceived depths of the monocular bar in different contexts are measured and compared.

Note that our manipulation of perceived relative depth position was not perfect: In Experiment 1, the monocular bar was perceived in front on only about two thirds of trials when it was presented later. If we had explicitly asked observers about the perceived relative depth position on each trial and only analysed those trials in which the monocular bar was perceptually in front, the effects in Experiment 3 would be expected to be larger. However, we opted not to use such a dual task paradigm, which would have increased the overall difficulty of the task. Not asking explicitly about the relative depth position also had the advantage that the context bar inducing the perceived differences in depth in Experiment 3 was completely irrelevant for the task.

What are the possible neural mechanisms through which filling-in affects stereoscopic depth perception? One possibility is that filling-in generates local occlusion cues, which determine relative depth positions and thus influence estimated depth from this early stage in a feed-forward manner. Neural activity of the monocular blind spot representation in V1 correlates with perceptual alternations during rivalry^37^, showing that filled-in information at the blind spot appears at an early level of the visual pathway. Another possibility is that a higher-level interpretation of relative depth position emerges and influences both blind spot filling-in and depth estimation from the top down. As neural representations of disparity information are widely distributed throughout different visual cortical areas^38^, the present experiments alone cannot determine at what stage filled-in information affects stereoscopic depth perception.

A number of studies have investigated the influence of occlusion on perceived depth. Nakayama & Shimojo^39^ studied “da Vinci stereopsis”, a common real-world scenario in which a surface occluding a more distant surface results in interocularly unpaired regions to its left and right. These unpaired monocular regions could produce depth signals and be perceived in the same depth plane as the more distant surface. Subsequent studies^40,41^ suggested that the perceived depth of these unpaired monocular regions could be explained by their roles as occlusion cues. Ramachandran & Cavanagh^42^ demonstrated that a subjective contour with a crossed disparity can give rise to a percept of occlusion, which can influence the matching of ambiguous repetitive elements in a stereogram – “stereoscopic capture”. Häkkinen & Nyman^43^ further showed that a phantom surface defined only by unpaired monocular cues (“da Vinci stereopsis”) could capture stereopsis of a surface of background dots that can be matched at multiple disparities. However, these studies cannot account for our results because we employed stimuli with unambiguous stereoscopic correspondence and disparity, and yet illusory occlusion with no retinal signal influenced depth estimates.

Stevenson & Koerding^44^ also showed that a potential occlusion relationship between two objects could provide structural constraints for the depth estimation of objects and therefore bias the perceived stereoscopic depth. Their account for structural constraints in depth estimation might explain how the visual system integrates the illusory relative depth position in the current paper. Nevertheless, our results demonstrate not only that occlusion can alter depth estimation defined by unambiguous binocular disparity, but also that this bias is robust even for illusory occlusion with no bottom up, retinal signal. It is likely that the probability of an illusory occlusion to be filled in could be modeled as cue reliability and be further integrated into standard probabilistic models^2–5^.

Other ordinal cues, like figure-ground assignments, integrate quantitatively with disparity, similar to what we observed for illusory occlusion^16–19^. In these studies, figure-ground organization was determined by convexity of contours. Burge *et al*.^18^ reported that different figure-ground organizations led to a difference of 0.056° disparity in estimating depth of the same disparity-defined region, while we show that illusory occlusion had a much larger effect (a difference of 0.27° disparity) on stereoscopic depth. Although this study and ours used foveal and peripheral stimuli, respectively, the difference in magnitude of influence might indicate that occlusion, even illusory, is a stronger cue to relative depth position than convexity^45^.

In summary, we demonstrated that a task-irrelevant illusory occluder could dominate over binocular disparity cues in the absence of retinal occlusion cues. The illusory occlusion internally generated by filling-in provided a perceived relative depth position that influenced metric stereoscopic depth estimates. Together with previous findings, we conclude that filling-in in the blind spot – often thought to be a low-level process with perceptual effects restricted to the blind spot area – produces opaque surface representations that are functionally integrated into other perceptual processes, such as depth estimation, affecting global object perception. These results may have implications for other situations in which visual information is perceptually filled-in, such as in the case of retinal scotoma.
